# Development and validation of a prediction model for cognitive impairment in elderly patients with type 2 diabetes

**DOI:** 10.3389/fnins.2026.1744871

**Published:** 2026-03-11

**Authors:** Sijie Li, Yan Jiang, Shanshan Liu, Ling Ke, Libingxue Huang, Xiangpeng Zhu, Qi Zhou

**Affiliations:** 1 General Department, Tianyou Hospital Affiliated to Wuhan University of Science and Technology, Wuhan, China; 2 Qingdao Mental Health Center, Qingdao, China

**Keywords:** cognitive impairment, elderly, risk prediction model, the old, type 2 diabetes

## Abstract

**Background:**

China is experiencing rapid population aging, accompanied by a rising prevalence of type 2 diabetes mellitus (T2DM) and its complex complications. Cognitive impairment is one of the major complications of T2DM and currently lacks effective treatment. These two conditions can interact and aggravate each other, forming a vicious cycle.

**Objective:**

This study aimed to identify reliable early predictors of cognitive impairment among elderly individuals with T2DM, in order to facilitate early intervention and delay disease progression.

**Methods:**

A total of 202 elderly patients with T2DM hospitalized at Tianyou Hospital, affiliated with Wuhan University of Science and Technology, between May and September 2025 were enrolled. Cognitive function was assessed using the Montreal Cognitive Assessment (MoCA) with a cutoff score of 26. Seventy-two participants scoring ≥26 were assigned to the normal cognition group, and 130 participants scoring ≤25 were assigned to the cognitive impairment group. Demographic information, hematological and imaging parameters, and scale scores related to sleep quality, anxiety–depression status, and activities of daily living were collected. Statistical analyses were conducted using R version 4.5.

**Results:**

Least absolute shrinkage and selection operator regression selected 14 predictors. After analyzing the data, four factors remained independently associated with T2DM related cognitive impairment: age (OR = 1.100, 95% CI: 1.038–1.164, *P* = 0.001), HADS-D score (OR = 1.242, 95% CI: 1.081–1.426, *P* = 0.002), WMD (OR = 2.444, 95% CI: 1.137–5.254, *P* = 0.022), and HbA1c (OR = 1.264, 95% CI: 1.007–1.585, *P* = 0.043). The model demonstrated an AUC of 0.812 (95% CI: 0.778–0.891) and was well-calibrated (Hosmer-Lemeshow *P* = 0.661). After bootstrap validation, the optimism-corrected AUC was 0.751, indicating minimal overfitting. At the optimal cut-off of 0.685, the model achieved a sensitivity of 69.2% and a specificity of 81.9%, with a positive predictive value of 87.4% and a negative predictive value of 59.6%. DCA demonstrated a positive net benefit across threshold probabilities from 0.02 to 0.86, supporting the model’s clinical value.

**Conclusion:**

This study developed a prediction model for T2DM related cognitive impairment in elderly Chinese patients. The model showed good discrimination, calibration, and clinical value, supporting its potential role for identifying high-risk populations. However, before using this model, more research is needed to confirm it’s performance in different people.

## 1 Introduction

Global population aging is accelerating, and the incidence of age-related diseases continues to rise annually. According to data from China’s seventh national population census, individuals aged 60 years and older account for 18.7% of the total population, representing approximately 260.4 million people (National Bureau of Statistics of China, and Office of the Leading Group for the Seventh National Population Census of the State Council., 2021). A cross-sectional study based on the diagnostic criteria of the American Diabetes Association reported that the overall prevalence of diabetes in China is about 12.8%, with the rate among individuals over 60 years old reaching approximately 30% (Li et al., 2020). Cognitive impairment refers to varying degrees of cognitive dysfunction caused by multiple factors, affecting abilities such as calculation, orientation, memory, and executive function. These deficits can impair daily living activities and, in severe cases, lead to death (Ni et al., 2022). Surveys have shown that the combined prevalence of mild cognitive impairment and dementia accounts for more than one-fifth of the population aged 60 years and above in China (Jia et al., 2020). Type 2 diabetes can adversely affect cognitive function, while cognitive decline, in turn, impairs glycemic control, forming a mutually reinforcing vicious cycle (Srikanth et al., 2020). These findings underscore the importance and necessity of cognitive assessment in patients with type 2 diabetes.

Current research on risk factors for T2DM related cognitive impairment is abundant but most focus on single dimensions. Studies vary in the variables, which leads to disparate findings that lack of systematic integretion. As a result, the findings of these studies can’t be used for clinical practice. By developing this research to solve the problem. Using data from hospital medical records, we systematically analysis multidimensional risk factors–including demographic characteristics, blood-related parameters, and imaging data–to develop a T2DM related cognitive impairment risk prediction model. We examined the performance of the model by Bootstrap validation, optimism correction, ROC analysis, calibration curve, and DCA. The model performs satisfactorily. This study aims to establish a predictive tool for cognitive impairment screening in the Chinese T2DM population that balances predictive performance with clinical operability, thereby informing stratified intervention strategies.

## 2 Subjects and methods

### 2.1 Subjects

A total of 202 elderly patients with type 2 diabetes mellitus (T2DM) who were hospitalized at Tianyou Hospital, affiliated with Wuhan University of Science and Technology, between May 2025 and September 2025 were enrolled in this study. Based on cognitive status, participants were divided into a normal cognition group (*n* = 72) and a cognitive impairment group (*n* = 130). Cognitive function was assessed using the Montreal Cognitive Assessment (MoCA), which evaluates domains including calculation, memory, language, and visuospatial abilities. The total MoCA score ranges from 0 to 30. A score of ≤25 indicated cognitive impairment, whereas a score of ≥26 denoted normal cognition. For participants with less than 12 years of education, one additional point was added to the total MoCA score.

The diagnostic criteria for diabetes were based on the standards of the American Diabetes Association (Chinese Diabetes Society, 2025): the presence of typical symptoms of diabetes (e.g., polydipsia, polyphagia, polyuria, unexplained weight loss) along with any of the following–fasting venous plasma glucose (FPG) ≥ 7.0 mmol/L, glycated hemoglobin (HbA1c) ≥ 6.5%, –confirmed the diagnosis. In the absence of typical symptoms, diagnosis required two abnormal test results from the same or two different time points based on FPG and/or HbA1c that met or exceeded the diagnostic thresholds. The final diagnosis of T2DM was confirmed by two or more endocrinologists after comprehensive evaluation and exclusion of type 1 diabetes and other specific types of diabetes. In addition, patients with a previously confirmed diagnosis of type 2 diabetes mellitus were also included.

Inclusion criteria: (1) Age between 60 and 85 years; (2) Meeting the diagnostic criteria for T2DM; (3) Ability to understand and complete the relevant scales; (4) No significant visual, auditory, or speech impairments; (5) Hospitalization for ≥3 days; (6) Voluntary participation in this study.

Exclusion criteria: (1) Presence of psychiatric disorders; (2) Severe visual, auditory, or language communication impairments; (3) Prior diagnosis of cognitive impairment and use of related medication; (4) Parkinson’s disease; (5) Critical conditions such as acute heart failure, acute myocardial infarction, acute cerebral hemorrhage, or acute cerebral infarction; (6) Presence of other systemic diseases; (7) Rheumatic or autoimmune diseases; (8) Major surgery within the past 3 months; (9) Acute infections; (10) Severe diabetic complications; (11) History of severe craniocerebral trauma; (12) Incomplete clinical data.

This study was reviewed and approved by the Ethics Committee of Tianyou Hospital, affiliated with Wuhan University of Science and Technology, and all participants provided informed consent prior to enrollment.

### 2.2 Methods

#### 2.2.1 Patient baseline data

Baseline demographic and clinical data collected from all participants included gender, age, educational level (categorized as illiterate, primary school, junior high school, high school or technical secondary school, and associate degree or above), duration of diabetes (years), living status (living alone: yes/no), smoking and alcohol consumption history (smoking only, alcohol consumption only, both smoking and alcohol consumption, or neither), and the presence of comorbidities, including hypertension (HTN), coronary atherosclerotic heart disease (CAD), and atrial fibrillation (AF) (all recorded as yes/no).

#### 2.2.2 Blood-related parameters

All participants fasted for at least 8 h overnight. Fasting venous blood samples were collected from the antecubital vein the following morning and sent to the hospital’s biochemistry laboratory for analysis. The measured parameters included neutrophil count, monocyte count, lymphocyte count, platelet count, uric acid, creatinine, albumin, fibrinogen, glycated hemoglobin (HbA1c), fasting plasma glucose, fasting triglycerides, and fasting high-density lipoprotein (HDL) cholesterol.

#### 2.2.3 Imaging data

(1) Carotid Plaque: Carotid color Doppler ultrasonography was conducted for all subjects by two experienced sonographers to evaluate the presence or absence of carotid plaques.

(2) Brain Imaging: Head CT and MRI scans were independently reviewed by two radiologists to determine the presence or absence of cerebral atrophy, white matter lesions (leukoaraiosis), and lacunar infarctions. All image interpretations were performed by senior neuroradiologists, and all reports complied with routine clinical diagnostic standards. Prior to data collection, all participating radiologists attended two standardized training sessions focused on the imaging criteria for brain atrophy, leukoaraiosis, and lacunar infarction. These sessions employed reference atlases to harmonize interpretation definitions and reduce inter-observer variability. Although a formal inter-rater reliability assessment was not conducted prior to the study, the combination of strict interpreter qualifications and pre-collection calibration training substantially minimized potential interpretation bias.

#### 2.2.4 Other relevant scales

(1) Pittsburgh Sleep Quality Index (PSQI) (Lu et al., 2014): Used to assess sleep quality over the past month. It includes components such as subjective sleep quality, sleep duration, sleep efficiency, and daytime dysfunction. The total score ranges from 0 to 21, with a higher score indicating poorer sleep quality.
(2) Hospital Anxiety and Depression Scale (HADS) (Sun et al., 2017): Used to screen for states of anxiety and depression. It contains 14 items, with 7 items each for the anxiety (HADS-A) and depression (HADS-D) subscales. Each subscale score ranges from 0 to 21, with a higher score indicating more severe anxiety or depressive symptoms.
(3) Barthel Index (BI) (Mlinac and Feng, 2016): Used to measure functional independence in activities of daily living. It consists of 10 items, including feeding, bathing, toileting, etc., with a total score ranging from 0 to 100. A higher score indicates a greater level of independence in daily activities.

#### 2.2.5 Formulas for research-related indicators

(1) Body Mass Index (BMI) = weight (kg)/height^2^ (m^2^). Height and weight were measured using standardized protocols.
(2) Platelet to High-Density Lipoprotein Ratio (PHR) = Platelet count (10^9^/L)/HDL-C (mg/dL). This ratio reflects inflammatory and prothrombotic states. A study involving a U.S. population suggested a non-linear negative correlation between PHR and cognitive function (Wang et al., 2024).
(3) Geriatric Nutritional Risk Index (GNRI) = [1.489 × serum albumin (g/L)] + [41.7 × (actual body weight/ideal body weight)]. Ideal body weight was calculated as follows (Bouillanne et al., 2005): Male Ideal Weight = [Height (cm) − 100] − [(Height (cm) − 150)/4]; Female Ideal Body Weight = [Height (cm) − 100] − [(Height (cm) − 150)/2.5]. Based on the score, nutritional risk was categorized into four levels: >98 indicates no nutritional risk; 92 ≤ GNRI ≤ 98 indicates low nutritional risk; 82 ≤ GNRI < 92 indicates moderate nutritional risk; and <82 indicates high nutritional risk. The GNRI provides a rapid and objective assessment of nutritional status in the elderly (Dai et al., 2024).
(4) Triglyceride-Glucose Index (TyG) = Ln [fasting triglycerides (mg/dL) × fasting glucose (mg/dL)/2], as a simple and relatively accurate surrogate marker for assessing insulin resistance (Kui et al., 2024).
(5) Aggregate Index of Systemic Inflammation (AISI) = [Neutrophil count (10^9^/L) × Platelet count (10^9^/L) × Monocyte count (10^9^/L)]/Lymphocyte count (10^9^/L).
(6) Systemic Inflammation Response Index (SIRI) = [Neutrophil count (10^9^/L) × Monocyte count (10^9^/L)]/Lymphocyte count (10^9^/L).
(7) Systemic Immune-Inflammation Index (SII) = [Platelet count (10^9^/L) × Neutrophil count (10^9^/L)]/Lymphocyte count (10^9^/L).
(8) Neutrophil to Lymphocyte Ratio (NLR) = Neutrophil count (10^9^/L)/Lymphocyte count (10^9^/L).
(9) Monocyte to Lymphocyte Ratio (MLR) = Monocyte count (10^9^/L)/Lymphocyte count (10^9^/L).
(10) Fibrinogen to Albumin Ratio (FAR) = Fibrinogen (g/L)/Albumin (g/L).

#### 2.2.6 Missing data handling

Patients with missing data for any candidate predictor were excluded during the data collection phase and did not enter the final analytic dataset (complete-case analysis). Only patients with complete data on all variables of interest were included in this study.

### 2.3 Statistical analysis

All data analyses were conducted using R software (version 4.5.x). Categorical variables were expressed as counts and percentages (*n*, %) and compared using the Chi-square (χ^2^) test. Ordinal variables were analyzed using non-parametric tests, with *post hoc* pairwise comparisons adjusted by the Bonferroni correction. Continuous variables were described as mean ± standard deviation (SD) for approximately normally distributed data and compared using the *t*-test; otherwise, they were reported as median (interquartile range, P25–P75) and compared using non-parametric tests.

All 44 candidate predictors were directly entered into a least absolute shrinkage and selection operator (LASSO) regression model with 10-fold cross-validation to select the most informative features while addressing multicollinearity and avoiding overfitting. No univariate pre-screening was performed to avoid potential selection bias. All continuous predictors were standardized to have a mean of 0 and a standard deviation of 1 prior to LASSO regression. Independent predictors were then determined using binary logistic regression analysis. Model calibration was assessed using the Hosmer–Lemeshow goodness-of-fit test, and discriminative ability was evaluated by receiver operating characteristic (ROC) curve analysis, with calculation of the area under the curve (AUC), sensitivity, and specificity. Calibration curves and decision curve analysis (DCA) were further applied to assess the model’s performance and clinical utility. A *p*-value < 0.05 was considered statistically significant.

The following R packages and functions were used: LASSO regression: glmnet (cv.glmnet, glmnet); predictors were standardized prior to LASSO; optimal λ selected via 10-fold cross-validation. Logistic regression: rms (lrm); nomogram plotted using nomogram. ROC analysis: pROC (roc, auc). Calibration: rms (calibrate, 1,000 bootstrap resamples); Hosmer–Lemeshow test: ResourceSelection (hoslem.test). Decision curve analysis: dcurves (dca).

## 3 Results

### 3.1 Univariate analysis

Variables including gender, age, white matter disease, lacunar infarction, cerebral atrophy, carotid plaque, living status (living alone or not), hypertension, coronary artery disease (CAD), atrial fibrillation (AF), educational level, duration of diabetes, history of smoking and alcohol consumption, body mass index (BMI), glycated hemoglobin (HbA1c), Barthel Index (BI), Pittsburgh Sleep Quality Index (PSQI), Hospital Anxiety and Depression Scale–Anxiety (HADS-A) and Depression (HADS-D) subscales, Aggregate Index of Systemic Inflammation (AISI), Systemic Immune-Inflammation Index (SII), Neutrophil-to-Lymphocyte Ratio (NLR), Monocyte-to-Lymphocyte Ratio (MLR), Systemic Inflammation Response Index (SIRI), Fibrinogen-to-Albumin Ratio (FAR), Geriatric Nutritional Risk Index (GNRI), Triglyceride-Glucose Index (TyG), Platelet-to-Hemoglobin Ratio (PHR), uric acid, creatinine, and albumin were compared between the two groups. The results are summarized in Table 1.

**TABLE 1:** Baseline characteristics and univariate analysis of factors associated with cognitive impairment in elderly patients with type 2 diabetes.

Characteristic		Without CI (72, 35.6%)	With CI (130, 64.4%)	*X^2^/z/t*	*p*
Sex	Male	40 (39.2%)	62 (60.8%)	1.146	0.284
	Female	32 (32%)	68 (68%)		
WMD	Yes	43 (28.9%)	106 (71.1%)	11.359	0.001
	No	29 (54.7%)	24 (45.3%)		
LI	Yes	62 (33.3%)	124 (66.7%)	5.464	0.069
	No	10 (62.5%)	6 (35.7%)		
Brain atrophy	Yes	60 (33.3%)	120 (66.7%)	3.845	0.085
	No	12 (54.5%)	10 (45.5%)		
Carotid artery plaque	Yes	60 (33.1%)	121 (66.7%)	4.722	0.053
	No	12 (57.1%)	9 (42.9%)		
Living alone	Yes	11 (42.3%)	15 (57.7%)	0.578	0.447
	No	61 (34.7%)	115 (65.3%)		
HTN	Yes	60 (35.3%)	110 (64.7%)	0.057	0.811
	No	12 (37.5%)	20 (62.5%)		
CAD	Yes	33 (34.4%)	63 (65.6%)	0.128	0.720
	No	39 (36.8%)	67 (63.2%)		
AF	Yes	1 (12.5%)	7 (87.5%)	1.036	0.309
	No	71 (36.6%)	123 (63.4%)		
Education	Illiterate	0	3 (100%)	−3.077	0.002
	Primary school	6 (22.2%)	21 (77.8%)		
	Junior high school	14 (25%)	42 (75%)		
	High school/technical secondary school	38 (44.2%)	48 (55.8%)		
	Associate degree or above	14 (46.7%)	16 (53.3%)		
History of smoking	Never	50 (33.8%)	98 (66.2%)	0.817	0.366
	Occasional	2 (40%)	3 (60%)		
	Daily	20 (40.8%)	29 (59.2%)		
History of alcohol consumption	Never	50 (31.1%)	111 (68.9%)	7.892	0.005
	Occasional	10 (47.6%)	11 (52.4%)		
	Daily	12 (60%)	8 (40%)		
Age		66 (62, 71.75)	72 (66, 77)	−4.315	<0.001
BMI		25.312 ± 3.039	24.206 ± 3.470	2.198	0.024
HbAlc		6.7 (6.2, 7.575)	7 (6.4, 8.225)	−2.066	0.039
Duration of diabetes		8 (2, 15.875)	10 (4, 17)	−1.169	0.243
BI		100 (100, 100)	100 (95, 100)	−3.264	0.001
GNRI		105.875 (102.786, 108.891)	102.973 (99.436, 108.444)	−3.036	0.002
TYG		8.899 (8.518, 9.405)	9.023 (8.409, 9.666)	−1.118	0.263
Creatinine		69.35 (59.4, 85.075)	71.8 (62.475, 93.225)	−1.338	0.181
Uric acid		323.65 (264.525, 370.550)	334.15 (271.275, 382.1)	−0.341	0.733
Albumin		43.55 (41.225, 45.5)	41.85 (39.3, 45.325)	−2.263	0.024
PSQI		8 (4.25, 12)	8 (5, 13)	−1.008	0.314
HADS-A		0 (0, 2)	0 (0, 2)	−1.015	0.310
HADS-D		2 (0, 4)	3 (2, 5)	−4.036	<0.001
AISI		163.966 (110.876, 250.184)	172.559 (109.87, 274.634)	−0.442	0.658
SII		445.87 (340.165, 603.949)	493.836 (304.974, 700.788)	−1.086	0.278
NLR		2.179 (1.829, 2.801)	2.408 (1.753, 3.088)	−1.350	0.177
MLR		0.23 (0.173, 0.273)	0.227 (0.183, 0.304)	−0.658	0.51
SIRI		0.839 (0.594, 1.098)	0.851 (0.613, 1.233)	−0.558	0.577
FAR		6.630 (5.843, 8.037)	6.949 (6.167, 7.94)	−1.066	0.287
PHR		4.483 (3.649, 5.416)	4.371 (3.227, 5.636)	−0.725	0.468

WMD, white matter disease; LI, lacunar infarction.

*Post hoc* pairwise comparisons with Bonferroni correction were performed for educational level. No statistically significant differences were found between the groups for educational level (all adjusted *P* > 0.005). For alcohol consumption, the distribution differed significantly between the two groups (*P* = 0.0057), with cognitively normal individuals showing lower alcohol consumption levels.

Univariate analysis revealed significant group differences in white matter disease, age, BMI, HbA1c, GNRI, albumin, HADS-D score, and BI score (*p* < 0.05). Specifically, the presence of white matter disease, advanced age, lower BMI, poorer glycemic control (higher HbA1c), lower albumin levels, nutritional risk (lower GNRI), more severe depressive symptoms (higher HADS-D score), and reduced functional independence (lower BI score) were identified as risk factors associated with cognitive impairment.

### 3.2 Variable selection for the predictive model

**FIGURE 1:**
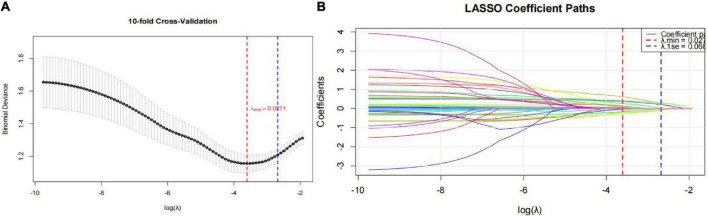
Screening of potential predictors based on LASSO regression. **(A)** 10-fold cross-validation; **(B)** coefficient profile paths.

The occurrence of cognitive impairment was set as the dependent variable. Cognitive impairment was designated as the dependent variable. All 44 candidate predictors were entered into a least absolute shrinkage and selection operator (LASSO) regression model (Figure 1A) to select the most informative features while addressing multicollinearity. Using 10-fold cross-validation, 14 variables with non-zero coefficients were identified. The cross-validation error plot is presented in Figure 1B. Notably, the univariate analysis presented in Table 1 focuses on the composite indices rather than their individual components. This approach was chosen because composite inflammatory markers have been shown to better reflect systemic inflammatory status. However, to comprehensively evaluate all potential predictors and avoid missing any informative signals, both individual cell counts and their derivatives were included in the LASSO regression for variable selection.

### 3.3 Development of the multivariable logistic regression model

The 14 variables selected by LASSO regression were included in a binary logistic regression. After analyzing the data, four factors remained independently associated with cognitive impairment: age, HADS-D, WMD, and HbA1c.

### 3.4 Development and validation of the nomogram prediction model

#### 3.4.1 Development of the nomogram

**FIGURE 2:**
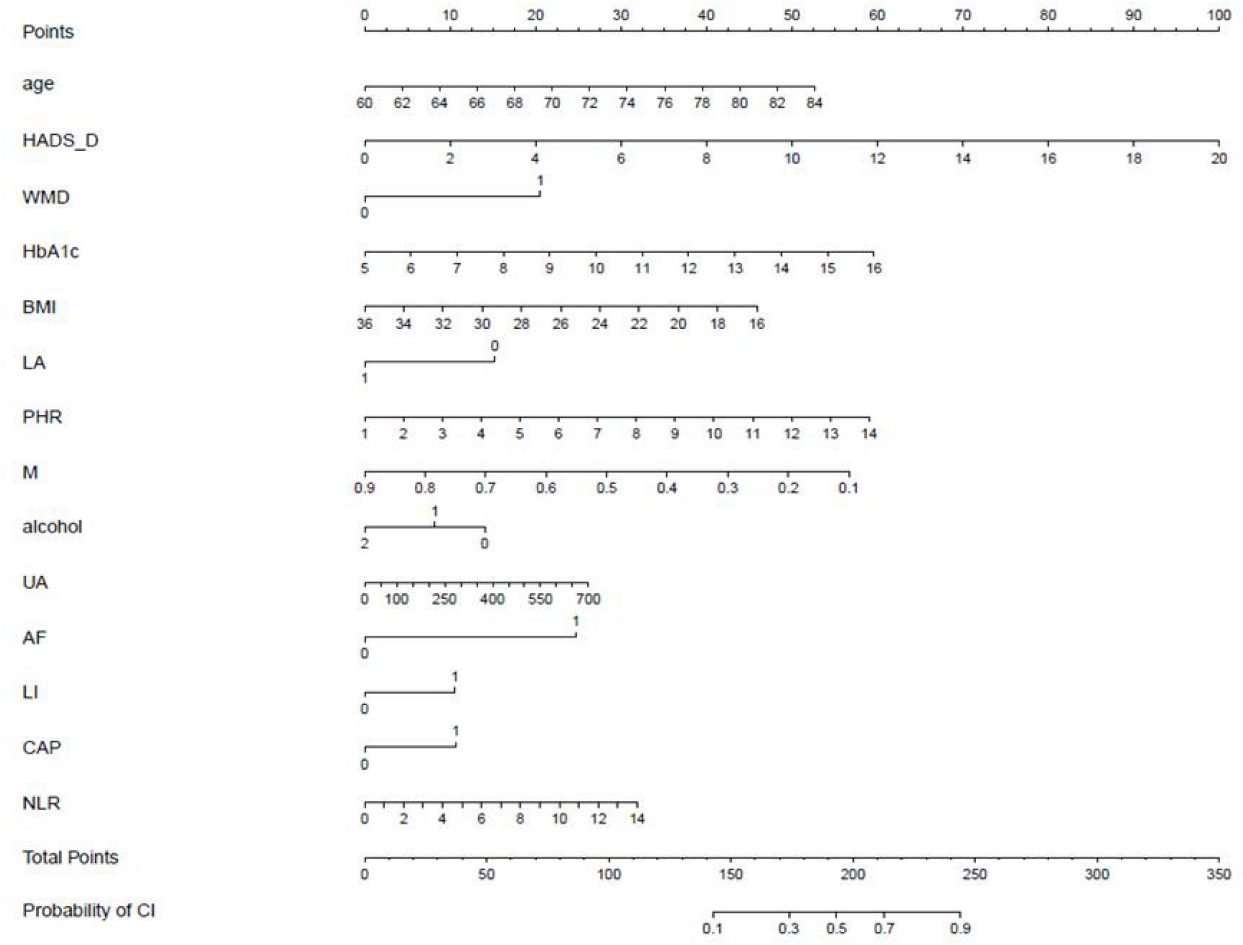
Nomogram for predicting the risk of cognitive impairment in the elderly population with type 2 diabetes. WMD, white matter disease; LA, living alone; M, Monocytes; alcohol, alcohol consumption; UA, Uric acid; AF, Atrial fibrillation; CAP, Carotid plaque; LI, Lacunar infarction.

A nomogram was constructed to represent the variables of the logistic regression model (Figure 2).

#### 3.4.2 The predictive value of the model was assessed

**FIGURE 3:**
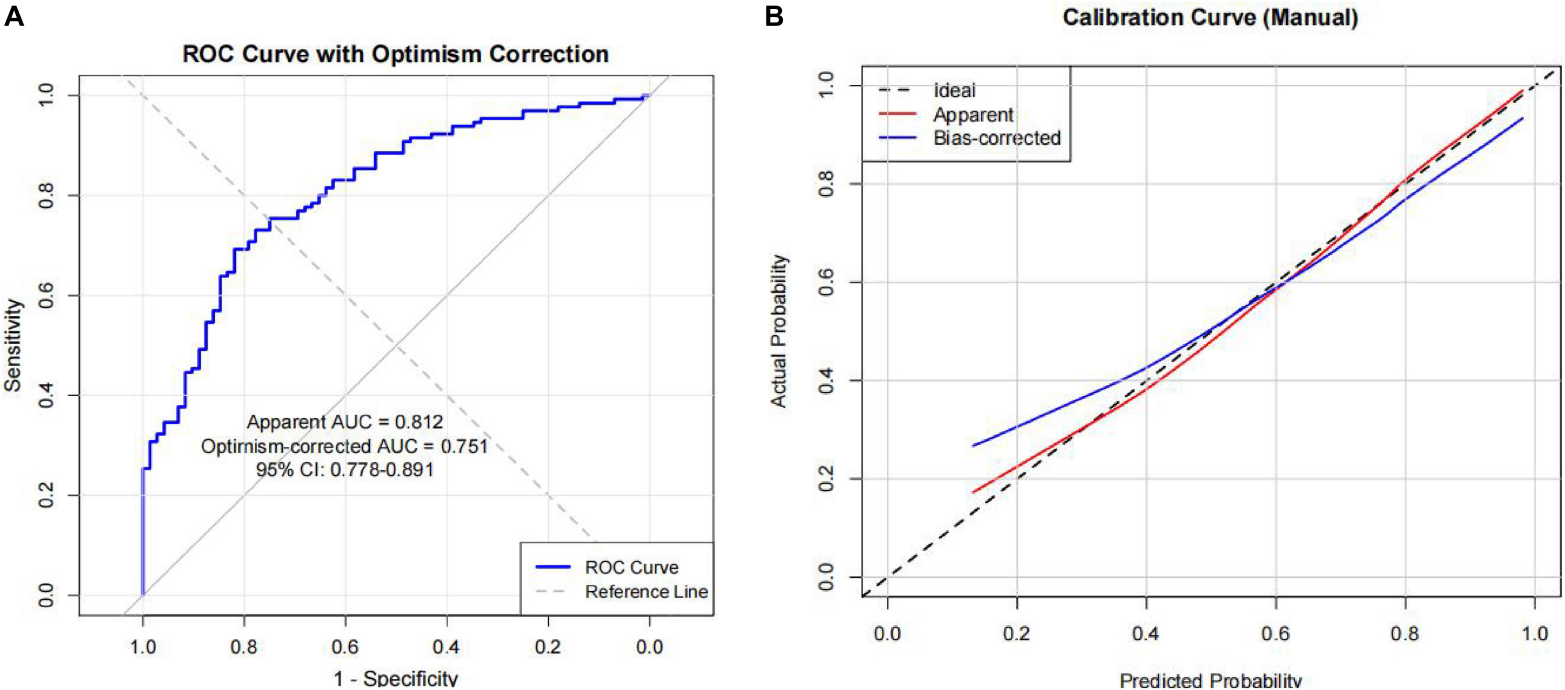
Receiver operating characteristic (ROC) curve **(A)** and calibration curve of the model **(B)**.

The ROC (Figure 3A) curve demonstrated an area under the curve (AUC) of 0.812 (95% CI: 0.778–0.891). Following 1,000 bootstrap resamples, the optimism-corrected AUC was 0.751. Using the optimal cut-off of 0.685, the model achieved a sensitivity of 69.2% and a specificity of 81.9%, with an overall accuracy of 73.8%. The positive and negative predictive values were 87.4% and 59.6%, respectively. The Hosmer-Lemeshow goodness-of-fit test (*χ^2^* = 5.88, *P* = 0.661) showed good. Calibration was further assessed using a calibration plot with 1,000 bootstrap iterations, which showed close alignment between the apparent and bias-corrected curves across the entire range of predicted probabilities, confirming good model calibration (Figure 3B).

### 3.5 Evaluation of clinical application value

**FIGURE 4:**
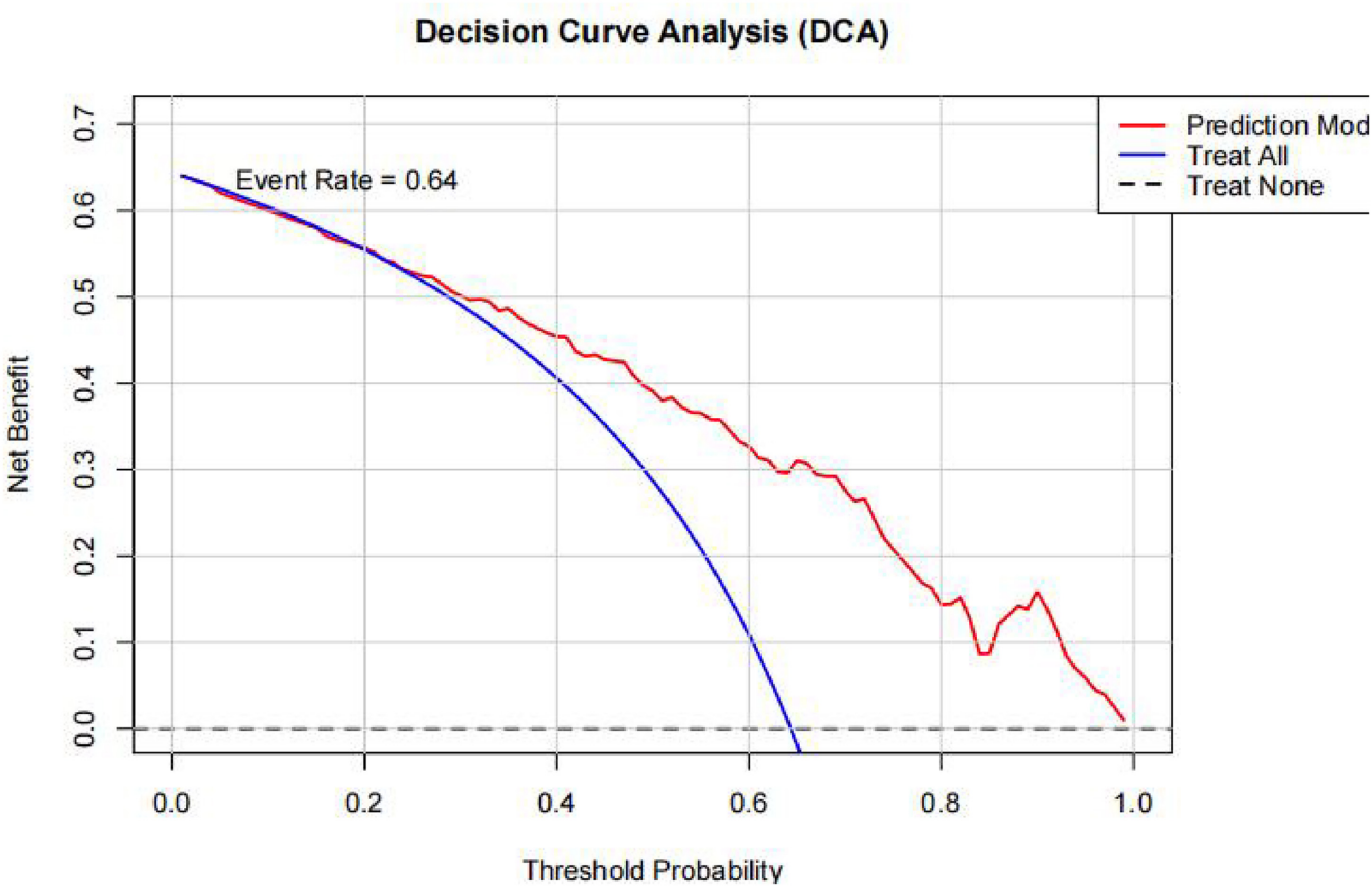
Decision curve analysis (DCA) of the mode.

The decision curve analysis (DCA) (Figure 4) is illustrated in the figure. DCA demonstrated that the prediction model provided a positive net benefit across a wide range of threshold probabilities (approximately 0.02–0.86), with net benefit values comparable to or exceeding those of the “treat all” strategy. This indicates that the model has clinical utility for guiding decisions regarding cognitive impairment risk assessment in patients with T2DM.

## 4 Discussion

This retrospective study employed the original Mandarin Chinese version of the Montreal Cognitive Assessment (MoCA, version 7.1) to evaluate patients’ cognitive function. Given the absence of validated cut-off values for this specific version, a score of 26–originally proposed for the English version–was adopted to distinguish cognitively normal individuals from those with cognitive impairment. At present, clinical management of cognitive impairment primarily aims to delay disease progression and enhance patients’ quality of life, as no long-term effective treatments are available (Rao et al., 2023). Therefore, early identification and prevention of cognitive impairment are of critical importance.

Recent investigations conducted in Western populations offer valuable context for interpreting our results. Drawing upon the extensive UK Biobank resource, Yang P. et al. (2025) developed a dementia risk assessment tool specifically for individuals with diabetes. Their analysis of 42,881 participants revealed that age, educational background, and glycemic control metrics consistently emerged as influential factors, with their final model attaining an AUC of 0.801 (Yang Y. et al., 2025). Parallel work by Wu et al. (2024), leveraging the nationally representative NHANES database, yielded a nomogram incorporating age, education, and uric acid that performed comparably (AUC: 0.786 in training, 0.777 in validation) among 878 older adults with diabetes. The robustness of these predictors across diverse populations was further reinforced by a recent machine learning study that utilized the NHANES III cohort for external validation. That study, which included 2,074 participants, identified education, age, BMI, and diabetes duration as key determinants of mild cognitive impairment in type 2 diabetes, achieving an AUC of 0.74 in internal validation and 0.80 upon external validation using the US-based dataset (Li et al., 2025). Beyond these cross-sectional insights, longitudinal evidence from the Mayo Clinic Study of Aging underscores the temporal relationship between glycemic dysregulation and cognitive trajectories, demonstrating that elevated HbA1c accelerates cognitive decline over more than 6 years of follow-up (Pink et al., 2025). While these international studies consistently underscore the importance of age, education, and glycemic markers–factors also prominent in our model–the present investigation extends current knowledge by identifying cerebral white matter disease and inflammatory indices (NLR, PHR) as additional determinants, potentially reflecting population-specific characteristics or the particular vulnerability of cerebrovascular integrity in Chinese diabetic patients. In this context, we conducted a retrospective analysis of 202 elderly patients with type 2 diabetes to further investigate these associations. A total of 202 elderly patients with type 2 diabetes were included in the study, among whom 130 (64.4%) were identified as having cognitive impairment. Univariate analysis revealed that the presence of cerebral white matter disease, advanced age, low body mass index (BMI), poor glycemic control, hypoalbuminemia, malnutrition, depressive symptoms, and lower Barthel Index (BI) scores were potential risk factors for cognitive impairment. All 44 candidate predictors were entered directly into a least absolute shrinkage and selection operator (LASSO) regression model to avoid potential selection bias associated with univariate pre-screening. Using 10-fold cross-validation, 14 variables with non-zero coefficients were selected and subsequently incorporated into a multivariable logistic regression model. After multivariable adjustment, four factors remained independently associated with cognitive impairment (Table 2): age (OR = 1.100, 95% CI: 1.038–1.164, *P* = 0.001) depressive symptoms (HADS-D score) (OR = 1.242, 95% CI: 1.081–1.426, *P* = 0.002), cerebral white matter disease (OR = 2.444, 95% CI: 1.137–5.254, *P* = 0.022), and HbA1c (OR = 1.264, 95% CI: 1.007–1.585, *P* = 0.043). Body mass index (BMI) demonstrated a borderline protective effect (OR = 0.906,95% CI: 0.812–1.011, *P* = 0.078), while monocytes (OR = 0.046,95% CI: 0.002–1.030, *P* = 0.052) and PHR (OR = 1.218,95% CI: 0.982–1.513, *P* = 0.073) showed trends toward association.

**TABLE 2:** Multivariate logistic regression analysis of cognitive impairment in the elderly population with type 2 diabetes.

Characteristic	*B*	Std. error	Wald *χ*^2^	*p*	*Exp (B)*	95% CI lower	95% CI upper
Age	0.095	0.029	10.520	0.001	1.100	1.038	1.164
HADS-D	0.216	0.071	9.368	0.002	1.242	1.081	1.426
WMD	0.894	0.390	5.243	0.022	2.444	1.137	5.254
HbAlc	0.234	0.116	4.098	0.043	1.264	1.007	1.585
BMI	−0.099	0.056	3.111	0.078	0.906	0.812	1.011
Living alone	−0.666	0.487	1.865	0.172	0.514	0.198	1.336
PHR	0.198	0.110	3.207	0.073	1.218	0.982	1.513
Monocytes	−3.071	1.582	3.769	0.052	0.046	0.002	1.030
Alcohol consumption	−0.296	0.275	1.161	0.281	0.744	0.434	1.274
Uric acid	0.002	0.002	0.734	0.391	1.002	0.998	–1.005
Atrial fibrillation	1.071	1.253	0.730	0.393	2.917	0.250	34.015
Carotid plaque	0.455	0.550	0.684	0.408	1.576	0.536	4.633
Lacunar infarction	0.466	0.667	0.488	0.485	1.593	0.431	5.892
NLR	0.099	0.150	0.436	0.509	1.104	0.823	1.480
Constant	−7.836	2.646	8.768	0.003	–	–	−7.836

Dependent variable coding: 1 = cognitive impairment, 0 = normal control. OR > 1 indicates risk factor.

Advanced age was identified as an independent risk factor for cognitive impairment (OR = 1.100, 95% CI: 1.038–1.164, *P* = 0.001), consistent with findings from a cross-sectional study in a Chinese population (Wang et al., 2022). This association may reflect age-related alterations in brain structure, neurophysiological function, and sensory processing (e.g., auditory and visual decline). However, our findings did not replicate associations with gender or education level reported in the same study, possibly due to the relatively small sample size in the present cohort.

The presence of cerebral white matter disease also emerged as an independent risk factor (OR = 2.444, 95% CI: 1.137–5.254, *P* = 0.022). Patients with white matter disease exhibited a 2.65-fold higher likelihood of cognitive impairment, a result consistent with meta-analytic evidence linking white matter lesions to cognitive decline (Hu et al., 2021).

In addition, higher HADS-D scores were independently and positively associated with cognitive impairment (OR = 1.242, 95% CI: 1.081–1.426, *P* = 0.002), in agreement with the findings of Yang P. et al. (2025), who similarly identified depressive symptoms as a significant predictor.

Elevated HbA1c remained as significant predictor (OR = 1.264) reinforcing the critical role of glycemic control in preserving cognitive function, as demonstrated in longitudinal studies. The borderline protective effect of BMI (OR = 0.906, *P* = 0.078) aligns with the “obesity paradox” observed in some elderly populations, although this association requires further investigation.

Overall, this study developed and internally validated a binary logistic regression model to predict cognitive impairment in elderly patients with type 2 diabetes. The model demonstrated good discrimination with an AUC of 0.812 (95% CI: 0.778–0.891) and was well-calibrated (Hosmer-Lemeshow *P* = 0.661; calibration plot mean absolute error = 0.038). After bootstrap validation, the optimism-corrected AUC was 0.751, indicating minimal overfitting. However, due to the insufficient sample size relative to the number of candidate predictors, this may lead to overfitting. Using the optimal cut-off value of 0.685, the model achieved a sensitivity of 69.2% and a specificity of 81.9%, with a positive predictive value of 87.4% and a negative predictive value of 59.6%. Decision curve analysis demonstrated a positive net benefit across threshold probabilities from 0.02 to 0.86, with net benefit values comparable to or exceeding those of the “treat all” strategy, supporting the model’s potential clinical utility.

Despite these strengths, this study has several limitations. The single-center, hospitalized population retrospective design may introduce selection bias, and the relatively small sample size (202 patients, 130 events) limited statistical power for some comparisons, as reflected in the wide confidence intervals for certain predictors. The use of a MoCA cutoff of 26, originally validated for the English version, may introduce outcome misclassification, as Mandarin-specific cutoffs have not been established. Although internal validation using bootstrap resampling was performed, external validation across diverse populations and settings is warranted to confirm the model’s generalizability. The modest negative predictive value (59.6%) suggests that the model may be more useful for ruling in high-risk individuals than for ruling out cognitive impairment. Future research should include larger, multi-center cohorts to refine the model and further enhance its predictive accuracy.

Despite these limitations, this study has several notable strengths. First, this study was based on data from hospitalized patients, which closely reflects clinical practice and enhances the clinical applicability of the findings. Second, we systematically integrated multidimensional risk factors–including demographic characteristics, blood-related parameters, and imaging data–providing a more comprehensive risk assessment framework for type 2 diabetes mellitus-related cognitive impairment. Third, internal validation using Bootstrap resampling was rigorously performed to assess model stability and overfitting. A nomogram was constructed to visualize the prediction model, thereby improving its operability in clinical decision-making. Finally, DCA demonstrated its clinical utility, supporting its potential application in routine diabetes care.

In conclusion, this study provides a clinically useful prediction model for type 2 diabetes mellitus-related cognitive impairment, supporting early screening and stratified intervention in Chinese patients with type 2 diabetes.

## Data Availability

The raw data supporting the conclusions of this article will be made available by the authors, without undue reservation.
