# Correction: Development and validation of a prediction model for cognitive impairment in elderly patients with type 2 diabetes

**DOI:** 10.3389/fnins.2026.1828964

**Published:** 2026-03-27

**Authors:** Sijie Li, Yan Jiang, Shanshan Liu, Ling Ke, Libingxue Huang, Xiangpeng Zhu, Qi Zhou

**Affiliations:** 1 General Department, Tianyou Hospital Affiliated to Wuhan University of Science and Technology, Wuhan, China; 2 Qingdao Mental Health Center, Qingdao, China

**Keywords:** cognitive impairment, elderly, risk prediction model, the old, type 2 diabetes

In the abstract, there was an error regarding the data presentation associated with Table 2. Although the numerical data in the abstract remain unchanged, the description has been revised to accurately reflect the corrected Table 2. This has been corrected to read:

**Results:** After analyzing the data, four factors remained independently associated with T2DM related cognitive impairment: age (OR = 1.100, 95% CI: 1.038–1.164, *P* = 0.001), HADS-D score (OR = 1.242, 95% CI: 1.081–1.426, P = 0.002), WMD (OR = 2.444, 95% CI: 1.137–5.254, *P* = 0.022), and HbA1c (OR = 1.264, 95% CI: 1.007–1.585, *P* = 0.043).

During production of the article, Table 2 was corrected. However, the corresponding text that referenced the data in Table 2 was inadvertently not updated to align with the corrected values.

A correction has been made to the section **4. Discussion**:

“After multivariable adjustment, four factors remained independently associated with cognitive impairment (Table 2): age (OR = 1.100, 95% CI: 1.038–1.164, *P* = 0.001) depressive symptoms (HADS-D score) (OR = 1.242, 95% CI: 1.081–1.426, *P* = 0.002), cerebral white matter disease (OR = 2.444, 95% CI: 1.137–5.254, *P* = 0.022), and HbA1c (OR = 1.264, 95% CI: 1.007–1.585, *P* = 0.043). Body mass index (BMI) demonstrated a borderline protective effect (OR = 0.906, 95% CI: 0.812–1.011, *P* = 0.078), while monocytes (OR = 0.046, 95% CI: 0.002–1.030, *P* = 0.052) and PHR (OR = 1.218, 95% CI: 0.982–1.513, *P* = 0.073) showed trends toward association.

Advanced age was identified as an independent risk factor for cognitive impairment (OR = 1.100, 95% CI: 1.038–1.164, *P* = 0.001), consistent with findings from a cross-sectional study in a Chinese population.

The presence of cerebral white matter disease also emerged as an independent risk factor (OR = 2.444, 95% CI: 1.137–5.254, *P* = 0.022).

In addition, higher HADS-D scores were independently and positively associated with cognitive impairment (OR = 1.242, 95% CI: 1.081–1.426, *P* = 0.002),

Elevated HbA1c remained as significant predictor (OR = 1.264) reinforcing the critical role of glycemic control in preserving cognitive function, as demonstrated in longitudinal studies. The borderline protective effect of BMI ((OR = 0.906, *P* = 0.078) aligns with the “obesity paradox” observed in some elderly populations, although this association requires further investigation.”

The original version of this article has been updated.

